# Congenital Duodenal Obstruction, Situs Inversus Totalis, and Gastric Perforation in a Neonate

**DOI:** 10.21699/jns.v5i4.463

**Published:** 2017-04-15

**Authors:** Rajat Piplani, Samir K Acharya, Deepak Bagga

**Affiliations:** Department of Pediatric Surgery, V.M.M.C & Safdarjang Hospital, New Delhi

**Keywords:** Situs inversus, Duodenal obstruction, Duodenal atresia, Gastric perforation

## Abstract

We report a rare case of incomplete congenital duodenal obstruction (Type 1 duodenal atresia) in association with situs inversus totalis presenting with gastric perforation in a neonate. The infantogram was suggestive of perforation with air under diaphragm along with dextrocardia. On exploration, a pin point perforation at fundus near lesser curvature along with situs inversus was noted. Primary closure of gastric perforation was done. Patient was then discharged on full breast feeds but was readmitted with intolerance to feeds and recurrent bilious vomiting. Further, upper GI contrast study revealed partial duodenal obstruction. On re-exploration, duodenal web with central aperture was seen and duodeno-duodenostomy was done.

## Case Report 

**Figure 1:**
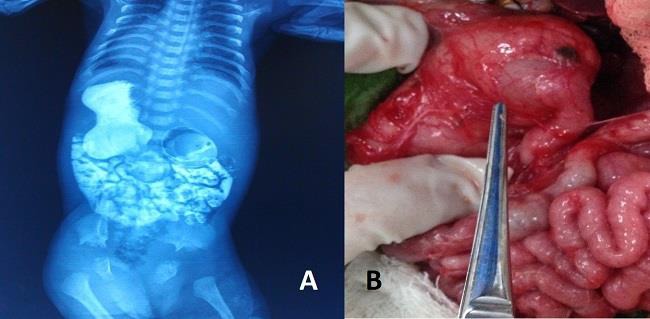
A) Contrast study showing partial duodenal obstruction and situs inversus. B) Operative figure showing duodenal obstruction.

A full term 2-day-old female neonate with birth weight 2 kg, born by normal vaginal delivery was referred with history of abdominal distension and recurrent bilious vomiting. The child had not passed meconium and had continuous bilious nasogastric aspirates. Examination revealed gross abdominal distension while on infantogram dextrocardia along with gas under both diaphragms was seen. On exploration, a pin point gastric perforation at fundus near lesser curvature was noted along with situs inversus. The stomach and spleen were found on right side while liver on left side of abdomen. There was incomplete gut rotation along with shortened mesentery. The stomach was normal sized and distal bowel was unused and small in calibre. The cecum and appendix were located in left lumbar region. Primary two layered closure of gastric perforation was done along with straightening of duodenum and widening of mesentery. In postoperative period, child passed stools and discharged on full breast feeds. But after two weeks, child was readmitted with intolerance to feeds and recurrent bilious vomiting. Initially managed with total parenteral nutrition and trial of feeds was subsequently started. The child was accepting feeds infrequently. Hence an upper GI contrast study was done which revealed partial duodenal obstruction (Fig.1A). Re-exploration confirmed duodenal web with central aperture between 2nd and 3rd part of duodenum (Fig.1B). A diamond shaped duodeno-duodenostomy was done. The child was kept on total parenteral nutrition and intravenous antibiotics. Postoperative period was uneventful and baby was discharged on full feeds on 7^th^ postoperative day.

## Discussion

The incidence of duodenal atresia is around 1 in 4000 to 1 in 15000 live births [1] and its concurrence with situs inversus totalis and gastric perforation is further rare. Congenital duodenal obstruction presenting as gastric perforation has been reported in only 13 cases in literature.[2,3] The association of situs inversus with congenital duodenal obstruction has been reported in about 20 cases in literature so far. [1] Among these, annular pancreas and duodenal web were commonly seen. However, pre-duodenal portal vein, duodenal atresia and duodenal stenosis have also been reported. [1] Situs inversus is usually asymptomatic and detected incidentally but when associated with congenital duodenal obstruction, it presents early in neonatal period.

Gastric perforation complicating congenital duodenal obstruction makes preoperative diagnosis difficult. Although the intestinal obstructions such as duodenal atresia, pyloric atresia, malrotation and annular pancreas are seldom the causes of neonatal gastric perforation, it should be explored during closure of perforation. [3] Both duodenal obstruction and nasogastric tube insertion were risk factors for gastric perforation in our case. The treatment of duodenal atresia with or without situs inversus remains duodeno-duodenostomy. [4] The outcome is also similar as the prognosis depends on associated cardiac anomalies. However, the surgical incision and mirror-image surgical anatomy can be taken care of, if the diagnosis is made preoperatively. In our case, we missed initial diagnosis of perforated duodenal web owing to incomplete duodenal obstruction. To conclude, congenital duodenal obstruction with situs inversus totalis is a rare association and presenting as neonatal gastric perforation is an extremely rare entity. Distal obstruction should be ruled out in neonates with gastric perforation. 

## Footnotes

**Source of Support:** None

**Conflict of Interest:** None
